# Clonal expansion and activation of tissue-resident memory-like T_H_17 cells expressing GM-CSF in the lungs of patients with severe COVID-19

**DOI:** 10.1126/sciimmunol.abf6692

**Published:** 2021-02-23

**Authors:** Yu Zhao, Christoph Kilian, Jan-Eric Turner, Lidia Bosurgi, Kevin Roedl, Patricia Bartsch, Ann-Christin Gnirck, Filippo Cortesi, Christoph Schultheiß, Malte Hellmig, Leon U.B. Enk, Fabian Hausmann, Alina Borchers, Milagros N. Wong, Hans-Joachim Paust, Francesco Siracusa, Nicola Scheibel, Marissa Herrmann, Elisa Rosati, Petra Bacher, Dominik Kylies, Dominik Jarczak, Marc Lütgehetmann, Susanne Pfefferle, Stefan Steurer, Julian Schulze zur Wiesch, Victor G. Puelles, Jan-Peter Sperhake, Marylyn M. Addo, Ansgar W. Lohse, Mascha Binder, Samuel Huber, Tobias B. Huber, Stefan Kluge, Stefan Bonn, Ulf Panzer, Nicola Gagliani, Christian F. Krebs

**Affiliations:** 1 III. Department of Medicine, Division of Translational Immunology, University Medical Center Hamburg-Eppendorf, Hamburg, Germany.; 2 Institute of Medical Systems Biology, University Medical Center Hamburg-Eppendorf, Hamburg, Germany.; 3 Hamburg Center for Translational Immunology (HCTI), University Medical Center Hamburg-Eppendorf, Hamburg, Germany.; 4 Center for Biomedical AI, University Medical Center Hamburg-Eppendorf, Hamburg, Germany.; 5 III. Department of Medicine, University Medical Center Hamburg-Eppendorf, Hamburg, Germany.; 6 I. Department of Medicine, University Medical Center Hamburg-Eppendorf, Hamburg, Germany.; 7 Protozoa Immunology, Bernhard Nocht Institute for Tropical Medicine, Hamburg, Germany.; 8 Department of Intensive Care Medicine, University Medical Center Hamburg-Eppendorf, Hamburg, Germany.; 9 Department of Internal Medicine IV, Oncology/Hematology, Martin-Luther-University Halle-Wittenberg, Halle (Saale), Germany.; 10 Institute of Clinical Molecular Biology, Christian-Albrechts University of Kiel, Kiel, Germany.; 11 Institute of Immunology, Christian-Albrechts-University of Kiel and UKSH Schleswig-Holstein, Kiel, Germany.; 12 Institute for Medical Microbiology, Virology and Hygiene, University Medical Center Hamburg-Eppendorf, Hamburg, Germany.; 13 Institute for Pathology, University Medical Center Hamburg-Eppendorf, Hamburg, Germany.; 14 Department of Legal Medicine, University Medical Center Hamburg-Eppendorf, Hamburg, Germany.; 15 I. Department of Medicine, Division of Infectious Diseases, University Medical Center Hamburg-Eppendorf, Hamburg, Germany.; 16 German Center for Infection Research, Partner Site Hamburg-Lübeck-Borstel-Riems, Hamburg, Germany.; 17 Department for General, Visceral and Thoracic Surgery, University Medical Center Hamburg-Eppendorf, Hamburg, Germany.; 18 Immunology and Allergy Unit, Department of Medicine, Solna, Karolinska Institute and University Hospital, Stockholm, Sweden.

## Abstract

Hyperinflammation contributes to lung injury and subsequent acute respiratory distress syndrome with high mortality in patients with severe coronavirus disease 2019 (COVID-19). To understand the underlying mechanisms involved in lung pathology, we investigated the role of the lung-specific immune response. We profiled immune cells in bronchoalveolar lavage fluid and blood collected from patients with severe COVID-19 and patients with bacterial pneumonia not associated with viral infection. By tracking T cell clones across tissues, we identified clonally expanded tissue-resident memory-like T_H_17 cells (T_RM_17 cells) in the lungs even after viral clearance. These T_RM_17 cells were characterized by a potentially pathogenic cytokine expression profile of *IL17A* and *CSF2* (GM-CSF). Interactome analysis suggests that T_RM_17 cells can interact with lung macrophages and cytotoxic CD8^+^ T cells, which have been associated with disease severity and lung damage. High IL-17A and GM-CSF protein levels in the serum of patients with COVID-19 were associated with a more severe clinical course. Collectively, our study suggests that pulmonary T_RM_17 cells are one potential orchestrator of the hyperinflammation in severe COVID-19.

## TH17 cells in severe COVID-19

Generation of T helper 17 (T_H_17) cells has been associated with immunopathogenesis in multiple autoimmune diseases. Using integrated single-cell transcriptome and TCR repertoire profiling, Zhao *et al*. showed that a population of T_H_17 cells with features of tissue-resident memory T cells was clonally expanded in bronchoalveolar lavage (BAL) fluid collected from the lungs of patients with severe COVID-19, but not in samples from patients with bacterial pneumonia. Lung tissue–resident memory-like T_H_17 cells were the primary immune cell type in BAL expressing the cytokine GM-CSF, which was also elevated in serum from a cohort of patients with severe COVID-19 compared with those with moderate disease. These results provide insight into specific T cell responses associated with severe COVID-19 pneumonia and identify a potential cellular target of GM-CSF–neutralizing therapies.

## Teaser

Tissue-resident memory-like T_H_17 cells are clonally expanded in bronchoalveolar lavage fluid of patients with severe COVID-19.

## INTRODUCTION

On 11 March 2020, the World Health Organization (WHO) communicated that the spread of the severe acute respiratory syndrome coronavirus 2 (SARS-CoV-2) had reached pandemic status. By the end of 2020, there were more than 80 million confirmed cases including 1.7 million deaths (*1*). These epidemiological data highlight the need to rapidly develop therapies for treating COVID-19 that reduce the high case fatality rate. The promising results of the clinical trial RECOVERY, in which dexamethasone was administered to 2104 patients with COVID-19 (*2*), suggest that one of the causes of the acute respiratory distress syndrome and ultimately death of patients with COVID-19 is the hyperactivation of the immune system. Supporting the pathogenic role of immune hyperactivation, the use of neutralizing antibodies, blocking, for example, granulocyte-macrophage colony-stimulating factor (GM-CSF) and interleukin-1β (IL-1β), has shown encouraging clinical results (*3*–*6*). The efficacy of a therapy blocking IL-6 has not yet been broadly recognized (*7*), but one recent study showed that tocilizumab reduced disease progression in patients with COVID-19 not receiving mechanical ventilation (*8*).

Considering that peripheral blood myeloid cells appear not to be able to produce high amounts of proinflammatory cytokines (*9*) and the numbers of blood T cells are reduced in patients with COVID-19 (*10*, *11*), the lungs may serve as a reservoir for cells producing these cytokines. However, additional investigation into the lung-specific cellular source of the proinflammatory cytokines typical of severe COVID-19, including IL-6, tumor necrosis factor–α (TNF-α), IL-1β, and IL-17A, is needed. Using single-cell RNA sequencing (scRNA-seq) methods, it has been shown that proinflammatory macrophages expressing *IL6*, *IL1B*, and *TNF* and CD8^+^ T cells with a tissue-resident cytotoxic signature are present in the bronchoalveolar lavage (BAL) and upper respiratory tract of patients with COVID-19 (*12*, *13*). The accumulation of interferon-γ (IFN-γ)–producing CD4^+^ T cells in the BAL of patients with COVID-19 has also recently been described (*14*). Still, the role of CD4^+^ T cells and of their different polarization states, especially at the site of infection, needs to be further elucidated.

CD4^+^ T cells orchestrate the immune response for example, by affecting on macrophage function and activation of cytotoxic CD8^+^ T cells. To mediate these different functions, naïve CD4^+^ T cells differentiate into effector cells characterized by different polarization states such as T helper cell 1 (T_H_1) and T_H_17. We have shown that T_H_17 cells can infiltrate the lungs and acquire a tissue-resident phenotype during bacterial infection (*15*). Upon infectious stimuli, these tissue-resident long-lived cells can reacquire the original cytokine profile, for example, IL-17A/F, or switch toward the production of IFN-γ, the signature cytokine of the T_H_1 polarization state. Although these long-lived tissue-resident T_H_17, referred to here as T_RM_17 cells, usually exert a protective function (*15*), we have also recently shown that these cells can contribute to immune-mediated inflammatory diseases (*16*). Whether T_RM_17 cells are present in the lungs of patients with COVID-19 and how they interact with other potentially pathogenic immune cells of these patients remains to be studied.

Here, we identified two populations of T_H_17 cells in the BAL fluid (BALF) of patients with COVID-19. One of these was mainly resident in the lung, characterized by the expression of GM-CSF and shared clones with T_H_1 cells. Moreover, we provide a lung-specific immune cell-cell interaction map showing the potential role of T_RM_17 cells in support of the already known pathological immune players, such as proinflammatory and profibrotic macrophages, and cytotoxic CD8^+^ T cells (*12*, *13*). These data provide support for continuing to test anticytokine therapies including those that have undergone preliminary clinical testing, for example, GM-CSF neutralization (*17*, *18*), or those now under consideration, for example, anti–IL-17A/F treatment (*19*).

## RESULTS

### Immune profile of T cells and myeloid cells in the blood and BALF of patients with COVID-19

**Fig. 1:**
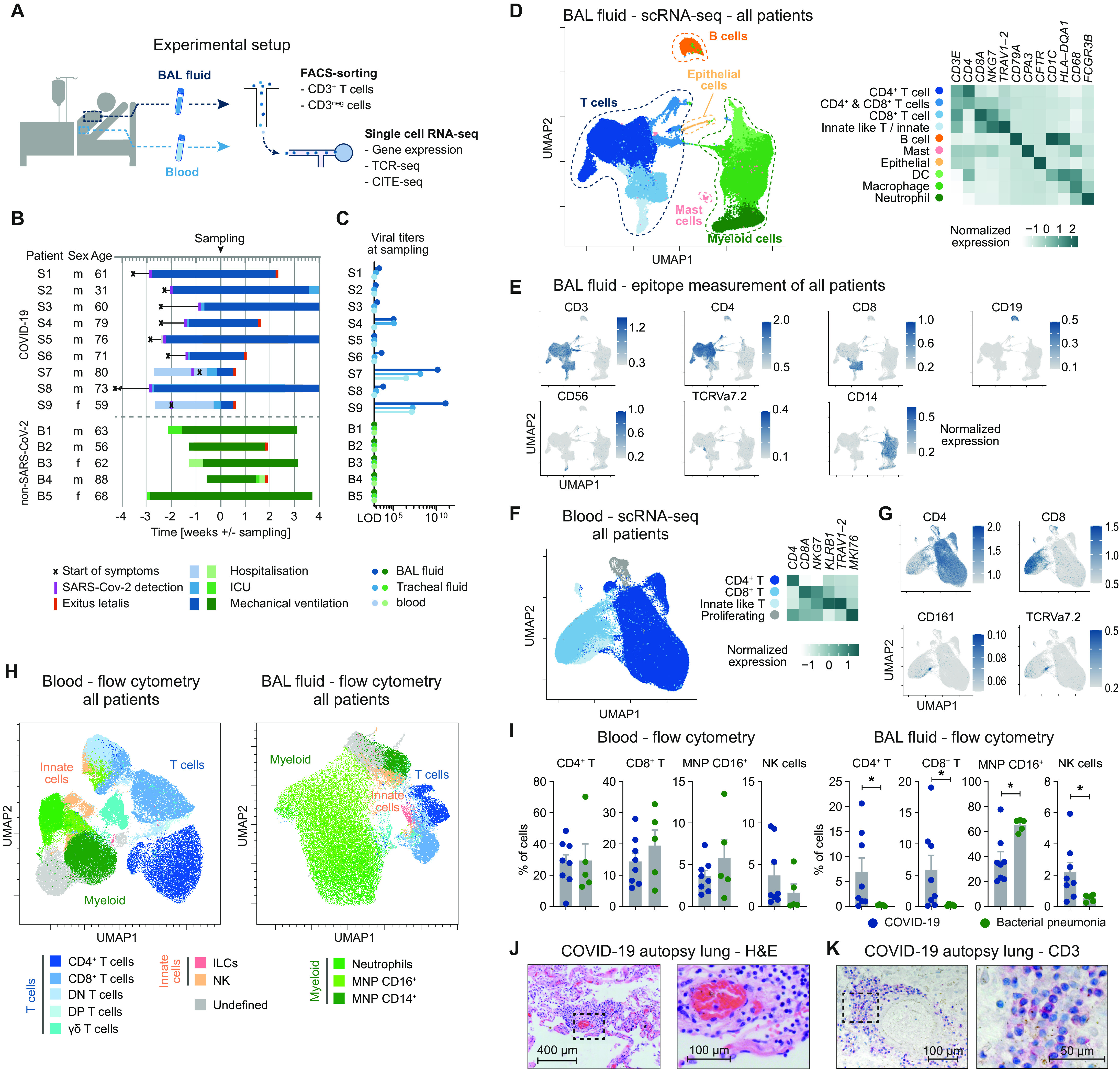
Immune landscape of severe COVID-19 and bacterial pneumonia. (**A**) Schematic representation of experimental setup. (**B**) Overview of baseline characteristics and clinical course of patients with COVID-19 and patients with bacterial pneumonia (LOD, limit of detection; ICU, intensive care unit). (**C**) Virus titers measured by quantitative polymerase chain reaction from BALF, tracheal fluid, and peripheral blood at time of sampling. (**D**) UMAP dimensionality reduction embedding of all cells from BALF (*n* = 56,735 cells, *n* = 8 for COVID-19, and *n* = 4 for bacterial pneumonia, samples of patients S6 and B1 were excluded for technical reasons) colored according to cell type assessed by gene expression and (**E**) epitope measurement using CITE-seq of key markers (scale bars indicate normalized expression). (**F**) Single-cell analysis of CD3^+^ T cells from peripheral blood of all patients (*n* = 77,457 cells, *n* = 7 for COVID-19, and *n* = 4 for bacterial pneumonia). (**G**) CITE-seq information of cluster-defining epitopes (scale bars indicate normalized expression). (**H**) Flow cytometry of peripheral blood and BALF of patients with COVID-19 (*n* = 8) and bacterial pneumonia (*n* = 5). Per patient, an equal number of viable CD45^+^ cells were exported for analysis and concatenated together before calculating the UMAP (total cells in peripheral blood = 129,141; in BALF = 114,927). Cell types were defined according to cell surface expression profiles by manual gating. Patient S9 was excluded from the statistical analysis due to low cell numbers. (**I**) Comparison of cell frequencies as measured by flow cytometry of cells from patients with COVID-19 and bacterial pneumonia (*P < 0.05). (**J**) Hematoxylin and eosin staining (H&E) and (**K**) CD3 staining of lung autopsy tissue of one representative of seven patients.

To provide a detailed analysis of the lung-specific and peripheral immune responses in COVID-19, BALF and peripheral blood were taken from nine patients with severe COVID-19. The major goal of the study was to analyze the tissue-specific immune response in patients with COVID-19, with a particular focus on T cells. In addition, we also included BALF and peripheral blood mononuclear cells (PBMCs) of five patients with bacterial pneumonia, not associated with viral infection. All samples were analyzed by flow cytometry and T cells were fluorescence-activated cell sorting (FACS)–sorted and subjected to scRNA and T cell receptor (TCR) sequencing, as well as to sequencing-based epitope measurement [cellular indexing of transcriptomes and epitopes by sequencing (CITE-seq)]. From BAL samples, CD3^−^ non–T cells, including mainly myeloid cells, were also analyzed by scRNA-seq and CITE-seq (56,735 cells from the BAL and 77,457 cells from the peripheral blood) (Fig. 1A and fig. S1, A and B).

All patients were treated on the intensive care unit at the University Medical Center Hamburg-Eppendorf. Eight of nine patients with COVID-19 and all patients with bacterial pneumonia were on mechanical ventilation at time of sampling, indicating the severity of disease, further reflected by high mortality in both groups (Fig. 1B). Detailed patient characteristics of patients with SARS-CoV-2 and patients with bacterial pneumonia, including comorbidities and relevant medications, are presented in table S1. In eight of the nine patients with COVID-19, symptomatic SARS-CoV-2 infection was diagnosed >2 weeks before BAL sampling, whereas in the remaining patients, SARS-CoV-2 was detected 8 days before sampling (Fig. 1B). In line with this, viral titers in BALF, tracheal secretion fluid, and blood of the majority of patients were very low or negative at time of sampling (Fig. 1C).

To examine the immune profile in the lungs of patients with COVID-19 and, at the same time, achieve robust clustering, we integrated the scRNA-seq datasets of patients with COVID-19 and bacterial pneumonia. The analysis revealed the presence of five main clusters in the BALF based on key signature genes and standard surface markers (T cells, B cells, mast cells, myeloid cells, and epithelial cells) represented in the Uniform Manifold Approximation and Projection (UMAP) dimension reduction (Fig. 1D). The T cell cluster could be further subdivided into CD4^+^ T cells CD8^+^ T cells, and innate-like T cells/innate lymphocytes (*NKG7* and *TRAV1-2*). Also, the myeloid cluster consisted of several subsets that were identified as macrophages (*CD68*), neutrophils (*FCGR3B*), and dendritic cells (DCs) (*HLA-DQA1*) (Fig. 1, D and E, and fig. S1C). All subsets identified were present in each sample of the two patient groups (fig. S1, D and E). We also combined RNA-, TCR-, and CITE-seq of peripheral blood T cells from patients with COVID-19 and bacterial pneumonia and yielded a sufficient number of CD4^+^, CD8^+^, and innate-like T cells for further clonal and expression analyses, allowing for a detailed comparison of peripheral and lung-specific T cell responses (Fig. 1, F and G, and fig. S2).

Next, we quantified the respective lymphoid and myeloid cell subsets in BALF and PBMC of patients with COVID-19 and bacterial pneumonia using flow cytometry data (Fig. 1H and fig. S3). Whereas in peripheral blood, there was not any obvious difference in the frequencies of cells analyzed comparing patients with COVID-19 and bacterial pneumonia, T cells subsets and natural killer (NK) cells showed significantly increased frequencies in the BALF of patients with COVID-19 compared with bacterial pneumonia (Fig. 1I). We confirmed the presence of T cells and mononuclear cells in the lung parenchyma of patients with COVID-19 by hematoxylin and eosin staining (Fig. 1J) and by CD3 staining (Fig. 1K) on lung autopsy tissue. An accumulation of T cells was predominantly identified in the perivascular space (fig. S4). Together, these data show an accumulation of T cells in the lungs of patients with COVID-19.

### Tracking T cell clonality in blood and BAL identifies tissue-specific T_RM_17 cells

**Fig. 2:**
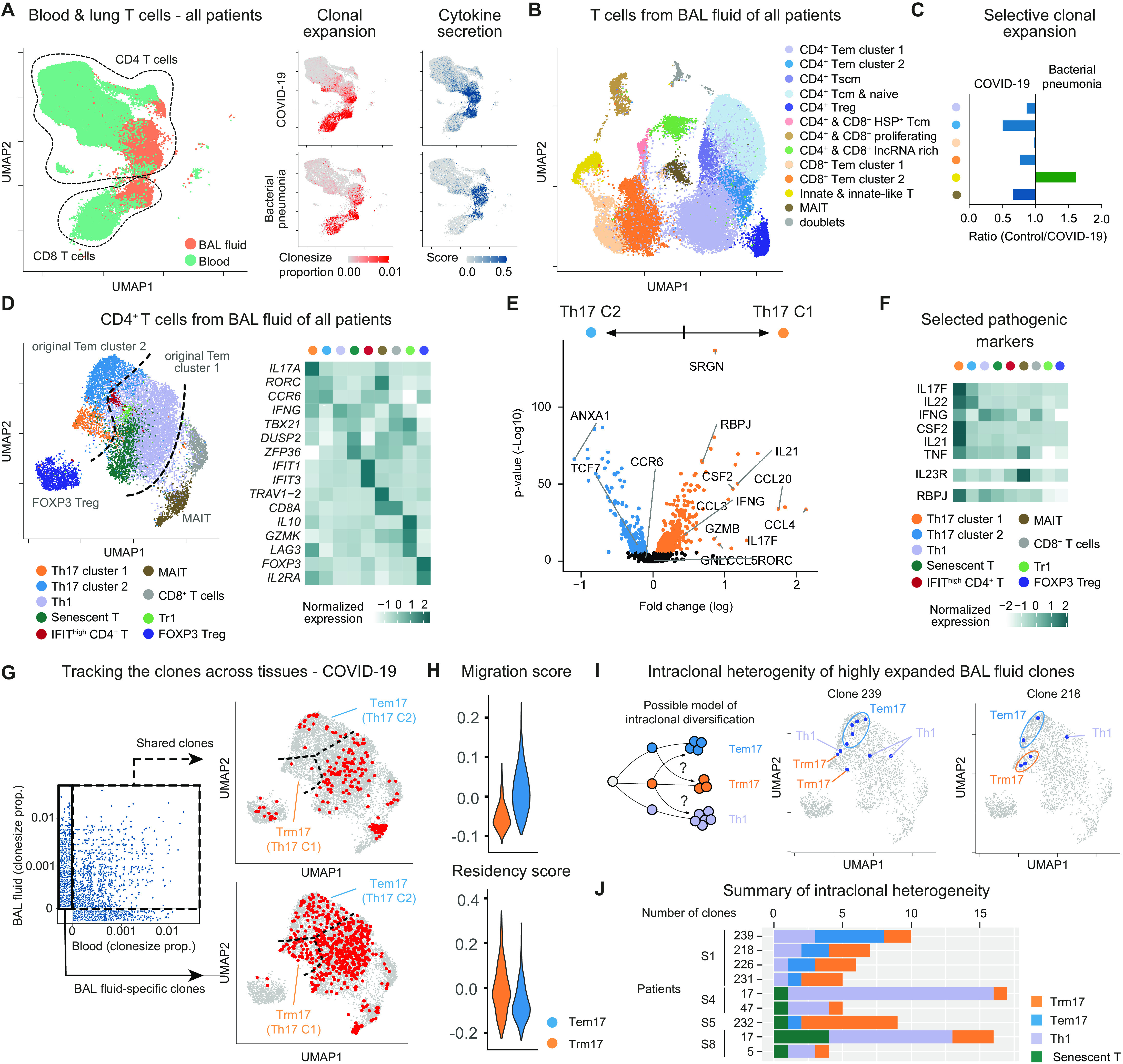
T cell clonality in pulmonary inflammation. (**A**) Blood-lung activation map of T cells from blood and BALF of all patients: UMAP dimensionality reduction embedding of T cells (left); clone size proportion (clone count divided by number of cells per sample) of T cells (middle), and the cytokine secretion score of T cells (right) from COVID-19 and bacterial pneumonia as indicated. (**B**) UMAP presentation of T cells from BALF of all patients. Clusters were annotated according to gene expression and epitopes measurement of key markers. T_CM_, T central memory; T_SCM_, T stem cell–like memory; lncRNA, long noncoding RNA. (**C**) Ratio of clonal expansion of bacterial pneumonia versus COVID-19 for the major expanded BALF T cell clusters. (**D**) Subclustering analysis of clonally expanded CD4^+^ T cells of all patients. Clusters were annotated according to gene expression presented in the heatmap. (**E**) Volcano plot showing differential gene expression between T_H_17 clusters 1 and 2 of all patients. Genes were considered significant with adjust *P* < 0.05. Nonsignificant genes are shown in black. (**F**) Heatmap of selected pathogenic gene markers of T_H_17 cells of all patients in comparison with other T cell clusters. (**G**) Clone size proportion of T cells in peripheral blood and BALF of patients with COVID-19 and presentation of high abundant clones (clone size > 5) that are shared between BALF and blood and BAL-specific clones as indicated. (**H**) CD4 migration and tissue residency score of T_H_17 cluster1 (T_RM_17) and 2 (T_EM_17) from all patients. (**I**) Possible model of intraclonal diversification of CD4^+^ T cell subsets (left); distribution of two representative BALF clones from a patient with COVID-19 (patient S1 clone239 and clone218) on the UMAP (middle and right). (**J**) Bar plot of top expanded BALF clones containing T_RM_17 cells from patients with COVID-19. COVID-19: *n* = 8 for BALF and *n* = 7 for blood; bacterial pneumonia: *n* = 4 for BALF and *n* = 4 for blood.

Considering the observation of the perivascular accumulation of T cells in the lungs of patients with COVID-19, we decided to further investigate the tissue-specific T cell response. We therefore integrated the blood and BALF T cell datasets to examine tissue-specific clonal expansion and activation of T cells. We examined T cell activation by analyzing the concomitant expression of a chosen pool of proinflammatory cytokines, i.e., *IL2*, *TNF*, *IL17A*, *IL17F*, *IFNG*, and *IL22*. Clonal expansion was analyzed by quantifying similar T cell clones on the basis of the TCR sequence information. We observed that both CD4^+^ and CD8^+^ T cells mainly underwent clonal expansion and activation predominantly in the BALF in both groups of patients (Fig. 2A). Clonal expansion does not necessarily reflect Ag specificity; therefore, we next investigated whether SARS-CoV-2–specific T cell clones are present in the BALF by comparing the TCRs identified in our study with those of two publicly available datasets of SARS-CoV-2–specific TCR sequences obtained from peripheral blood (*25*, *26*). The frequency of shared clones was higher in the COVID-19 cohort compared with the patients with bacterial pneumonia (fig. S6A). In addition, we observed higher frequencies of shared clones in T cells from the BALF as compared with peripheral blood.

Considering this tissue-specific activation, we examined BALF T cells with more granularity. By combining CITE-seq and differentially expressed genes (DEG), we identified five major CD4^+^ T cell clusters, including a population of Foxp3^+^ regulatory T (T_reg_) cells and two CD8^+^ T cells clusters (Fig. 2B and fig. S5). We also found three clusters composed of both CD4^+^ and CD8^+^ T cells and characterized by heat shock proteins, genes associated with proliferation (*MKI67* and *STMN1*) and expression of long noncoding RNAs (*MALAT1* and *NEAT1*). Last, we identified a distinct cluster formed by MAIT cells (TCRVa7.2/*TRAV1-2*) and one by innate and innate-like T cells (CD56 and *NKG7*).

We then quantified the clonal expansion for each population (fig. S6, B and C). We observed high clonal expansion in two CD4^+^ T cell clusters [i.e., CD4^+^ T effector memory (T_EM_) cells, clusters 1 and 2], in the two main CD8^+^ T cell populations, in MAIT cells, and in the other innate-like T cell cluster. We next wondered whether these cells were also expanded in a different type of infection. By comparing the clone size proportion of the above indicated populations between the two patient groups (Fig. 2C), we found that the CD4^+^ T_EM_ cell cluster 2 was most selectively expanded in patients with COVID-19 compared with patients with bacterial pneumonia. Therefore, we decided to further analyze this cluster and, as controls, we used the other CD4^+^ T_EM_ cell cluster (i.e., cluster 1), MAIT, and Foxp3^+^ T_reg_ cells (Fig. 2D and fig. S7). We observed that the original cluster 2 was enriched for genes typical of T_H_17 polarization states, whereas the original cluster 1 primarily contained CD4^+^ T_EM_ cells expressing genes associated with a T_H_1 polarization state. We then tested which of these clusters were selectively expanded in patients with COVID-19 and found that although T_H_1 cells are expanded in all patients, both T_H_17 clusters were only expanded in patients with COVID-19 (fig. S7, E and F).

Differential expression analysis revealed that although both T_H_17 clusters express similar levels of *RORC* and *CCR6*, T_H_17 cell cluster 1 is enriched for genes associated with cytotoxicity (*SRGN*, *GZMB*, and *GNLY*) and for genes translating for proinflammatory cytokines (*IL21*, *IL17F*, *IFNG*, and *CSF2/*GM-CSF) and chemokines (*CCL3*, *CCL4*, and *CCL5*) (Fig. 2E and fig. S7G). We also observed that this cluster has high expression of the transcriptional factor *RBPJ*, which has been shown to be fundamental for the pathogenicity of T_H_17 cells in an experimental autoimmune encephalomyelitis mouse model (*20*). We next compared the expression of some of these DEG, in addition to other genes associated with T_H_17 cell pathogenicity, among all the subclusters of the CD4^+^ T cells isolated from the BALF. We found that among all, *CSF2* (GM-CSF) and *IL21* were the most selective genes expressed by the cluster of the potentially pathogenic T_H_17 cells (Fig. 2F).

We have recently shown that T_H_17 cells can acquire a tissue-resident phenotype in the lungs (*15*). We therefore used TCR sequences as markers to test whether this population of T_H_17 cells is mainly found in the BALF and not in the circulation, suggesting possible resident behavior. We found that, virtually, none of the highly expanded T_H_17 cluster 1 cells in the lungs shared clones with T cells in the blood, supporting the notion that these cells are resident in the lung. In contrast, the other T_H_17 cell cluster (i.e., cluster 2) and the T_H_1 cell populations are composed of a mixture of resident and circulatory clones (Fig. 2G). Then, we used a literature-based residency and migratory scores and found that the potentially pathogenic T_H_17 cell cluster 1 has on average a lower migratory and higher residency score compared with the other effector T_H_17 cell cluster 2 (Fig. 2H). These data suggest that the T_H_17 cell cluster 1 is enriched for pathogenic and resident cells compared with the T_H_17 cell cluster 2. To simplify the classification of these clusters and reflect their features, we named cluster 1 as tissue-resident memory-like T_H_17 cells (T_RM_17) and cluster 2 as effector memory T_H_17 cells (T_EM_17). We observed that the TCR sequences shown to be specific for SARS-CoV-2 in other studies (*21*, *22*) can also be identified in the cluster of T_RM_17 cells (fig. S7H). Because it has been shown that T_RM_17 cells still retain a certain degree of plasticity, especially toward T_H_1 cells (*23*), we wondered whether this was the case in COVID-19. We used the TCR sequences as natural lineage barcodes to follow the origin/fate of some of the T_RM_17 cell clones expanded in patients with COVID-19. We observed that sister clones of the T_RM_17 cells were also found to express other T cell phenotypes, such as the T_H_1 phenotype (Fig. 2, I and J). This intraclonal diversification (*24*) suggests that some of the T_RM_17 cells have a dynamic developmental trajectory in common with other types of tissue-specific CD4^+^ T cell populations, in particular with T_H_1 cells that, as expected, are the dominant expanded clones in term of quantity. In summary, we identified lung-specific T_RM_17 cells in the BALF of patients with COVID-19 on the basis of cytokine expression profiles and clonal expansion.

### Different types of myeloid cells identified in the BALF of COVID-19

**Fig. 3:**
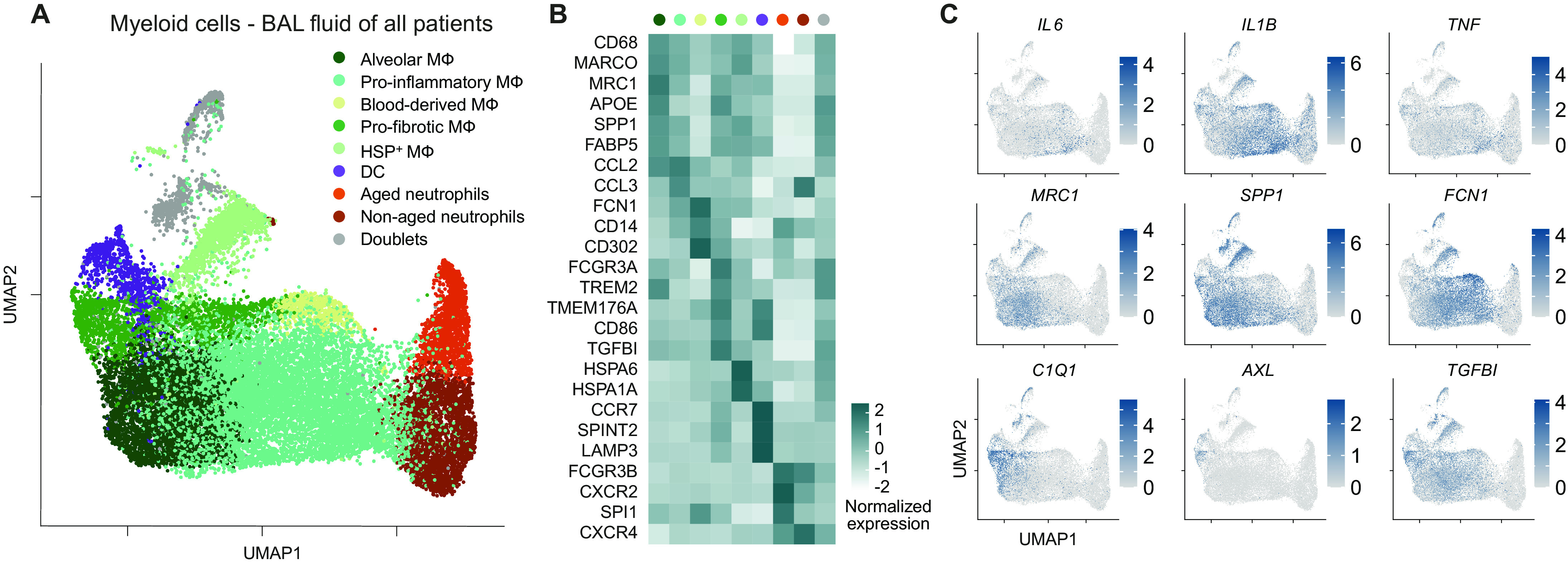
Landscape of myeloid cells in the lung. (**A**) UMAP dimensionality reduction embedding of myeloid cells from BALF of all patients from our study (COVID-19 *n* = 8 and bacterial pneumonia *n* = 4). (**B**) Heatmap of key marker gene expression of the indicated clusters. (**C**) UMAP plots showing expression of genes mirroring key features of macrophage polarization and function (scale bars indicate normalized expression).

Next, we set out to examine the different populations of myeloid cells in BALF that were identified in our scRNA-seq analysis (Fig. 1D). As above, to achieve robust clustering, we included myeloid cells from all patients (with COVID-19 and bacterial pneumonia) in this analysis. Subclustering of macrophages and neutrophils revealed the heterogeneity of macrophage polarization status and stages in neutrophil maturation (Fig. 3A). In particular, alveolar macrophages were defined by the gene expression of class A scavenger receptor *MARCO*, the mannose receptor *MRC1*, and the intracellular lipid transporter *FABP5*. They also express high levels of the profibrotic gene *SPP1* (Fig. 3, B and C) (*12*). We also detected high levels of *TREM2*, a surface receptor able to prevent macrophage apoptosis upon viral replication (*25*). Next, we defined proinflammatory macrophages as cells expressing high levels of *CCL2* and *CCL3*, chemokines involved in recruitment of adaptive and innate cells to sites of infection, and which were also characterized by the expression *IL6*, *IL1B*, and *TNF* (Fig. 3, A and C). Blood-derived macrophages have been defined on the basis of high expression of *CD14*. Their proinflammatory signature is mirrored by the high expression of *FCN1*, as previously described in patients with COVID-19 (*12*), by the high expression of *CD302*, a C-type lectin receptor induced in vitro upon lipopolysaccharide stimulation, and by the expression of alarmins such as *S100A8* and *S100A12*, calcium-binding proteins, and danger-associated molecular patterns (DAMPs), whose expression is regulated by proinflammatory molecules such as IFN-γ and TNF-α, and that can lead to the secretion of IL-6 and IL-8 (Fig. 3B and fig. S8A).

We defined a population of cells with a tissue-remodeling signature as profibrotic macrophages, which, under a persistent inflammatory trigger, might acquire a profibrotic function (*26*). These profibrotic macrophages expressed higher levels of *APOE*, *TGFBI*, *TMEM176A*, and *CD86* and were enriched in complement components (*C1QB*, *C1QA*, and *C1QC*) (Fig. 3, B and C, and fig. S8A), in line with what was previously described for profibrotic macrophages in the context of SARS-CoV-2 infection (*12*). This macrophage cluster showed a profibrotic signature mostly similar to the alveolar macrophage subcluster, potentially indicating that the two macrophage populations have a similar biological function. However, the profibrotic macrophages were also characterized by the expression of high levels of *FCGR3A* (CD16) and intermediate/low levels of *CD14*, together with the expression of genes associated with antigen presentation (fig. S8A), therefore suggesting them as a subcluster of cells potentially originating from a nonclassical/intermediate monocyte population. Profibrotic macrophages also expressed *AXL*, a receptor tyrosine kinase that is required for resolution of lung inflammatory disease upon viral infection, induced by GM-CSF, and associated with development of tissue fibrosis in mouse models (*27*, *28*). A population highly enriched in heat shock protein (*HSPA6*, *HSPA1A*, *HSPH1*, *DNAJB1*, and *HSPA1B*) was also observed and named as heat shock protein positive (HSP^+^) macrophages.

Last, expression of *CXCR2* and *CXCR4*, which define neutrophil maturation stages and regulate their trafficking from bone marrow, was detected in the two neutrophil clusters identified in patients with COVID-19 (aged and nonaged neutrophils) (Fig. 3, A and B). All subsets described were reproducibly found in both patients with COVID-19 and bacterial pneumonia (fig. S8, B and C). The identification of myeloid cell populations in our dataset provided us with a foundation for investigating the interactions between immune cells in the lungs of patients with COVID-19.

### T_RM_17 cell interactome with other pathological cell types in COVID-19

Once the landscape of the myeloid and lymphoid compartment was clarified, we investigated the cell-cell interactions of T_RM_17 cells with the other immune cells, in particular myeloid and cytotoxic CD8^+^ T cells, which are known to correlate with lung damage in patients with COVID-19 (*12*, *13*).

**Fig. 4:**
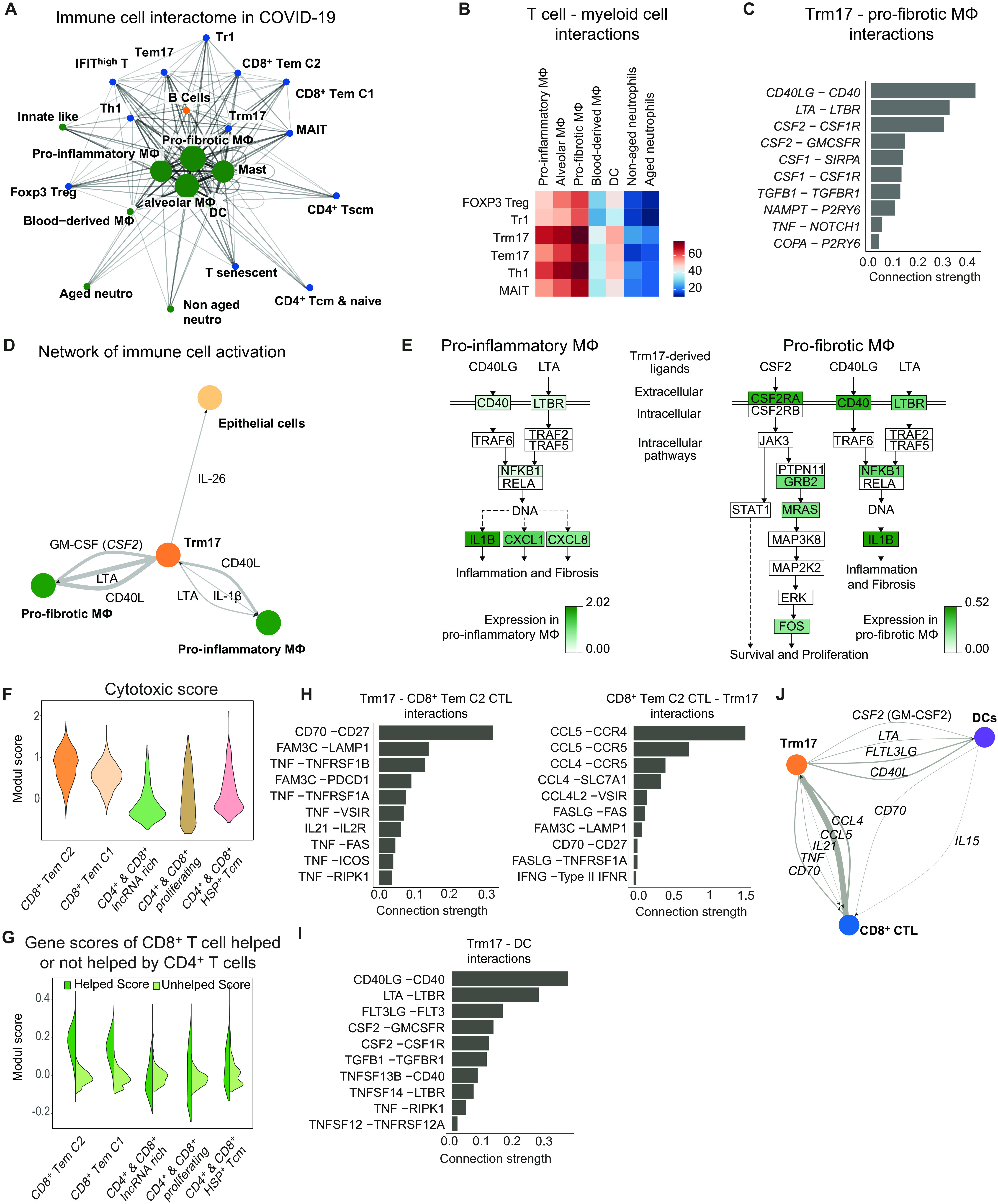
Interactome of T cells and myeloid cells in the lungs of patients with COVID-19. (**A**) Interaction network of all BALF clusters based on the number of ligand-receptor interaction (>30 edges) based on Fruchterman-Reingold force-directed algorithm from patients with COVID-19 (*n* = 8). (**B**) Adjacency map of T cell-myeloid cell interactions. (**C**) Ligand and receptor interaction strength ([mean ligand expression] × [proportion of receptor expression per cluster]) of T_RM_17 cells (ligands) and profibrotic macrophages (receptors). Interactions were filtered for cytokines and, for specificity, based on rank scoring. (**D**) Supervised interaction map of potential key players in sustaining lung inflammation in patients with COVID-19. Line width correlates with interaction strength. (**E**) Pathway analysis of CD40L (CD40LG), LTA, and GM-SCF (CSF2) signaling in proinflammatory and profibrotic macrophages indicating the log_2_ fold change in COVID-19 versus bacterial pneumonia. (**F**) Cytotoxic module scores in all clusters which include CD8^+^ cells using proinflammatory and cytotoxic mediator genes in CTL from (*13*). (**G**) Module scores in the indicated clusters using the highest 50 differential expressed genes of CD8^+^ T cells receiving help or no help from CD4^+^ cells, respectively, according to (*31*). (**H** and **I**) Ligands and receptor interaction strength (H) between T_RM_17 and CD8^+^ T_EM_ CTL Cluster 2 and (I) between T_RM_17 and DC. Interactions were filtered according to their rank score. (**J**) Supervised interaction map of T_RM_17, CD8^+^ T_EM_ CTL, and DCs with annotated ligands. Line width correlates with interaction strength.

We constructed the interactome of all immune cells found in the BALF of patients with COVID-19 using the T cell and the myeloid cell subclusters defined in Figs. 2 and 3, respectively. Then, we performed a network analysis that is based on transcriptomic levels of ligand-receptor interactions between cell types. We identified different clusters of macrophages (proinflammatory, profibrotic, and alveolar macrophages) at the center of our communication network, having the highest number of different ligand-receptor pairs with other cell populations (Fig. 4A). We also observed that the cells of the main T cell populations (T_H_1, T_RM_17, T_EM_17, FOXP3 T_reg_, Tr1, and MAIT) interact more with tissue macrophages than with other myeloid cell clusters, such as blood-derived macrophages, DCs, or neutrophils (Fig. 4B). Among the T cells, T_RM_17, T_EM_17, and T_H_1 appear to have more ligand/receptor interactions with macrophages than the other T cell clusters do (Fig. 4, A and B). To further explore the interactions between lung T_RM_17 that we previously found to be clonally expanded and macrophages, we selected the 10 most specific ligand-receptor pairs of both populations on the basis of rank calculated using CellPhoneDB (*29*). To calculate the connection strength of these interactions, we multiplied the average ligand expression with the proportion of cells expressing the receptor from the respective clusters. We found that CD40LG/CD40, LTA/LTBR (lymphotoxin-alpha/lymphotoxin-beta-receptor), and GM-CSF/GM-CSFR had the highest connection strength between T_RM_17 and profibrotic macrophages (Fig. 4C). In addition, we identified CD40LG/CD40, LTA-LTBR, and CSF2-CSF1R to demonstrate the most selective and strongest interactions of T_RM_17 with proinflammatory macrophages (fig. S9A).

To understand how T_RM_17 cells directly interact with the lung epithelium, we analyzed the interactions of T_RM_17 cells with epithelial cells. Here, we identified the cytokines IL-26, IL-17A, and IL-17F to be among the top 10 interactions (fig. S9B). IL-26 interacts with the IL-26R on epithelial cells (fig. S9C), potentially playing a role in antiviral response (*30*). Because of the low number of epithelial cells in our dataset, we confirmed the expression of IL-26R, a heterodimer consisting of IL-10RB and IL-20RA, using the transcriptomic data on epithelial cells recently published in the context of SARS-CoV-2 infection (*13*) (fig. S9D).

Then, focusing on the most relevant interactions on the basis of rank and connection strength between T_RM_17 cells, profibrotic macrophages, proinflammatory macrophages, and epithelial cells, we constructed a smaller curated interaction map that depicts that T_RM_17 can act on both profibrotic and proinflammatory macrophages as well as epithelial cells (Fig. 4D). In return, proinflammatory macrophages, by secreting IL-1β, may act on T_RM_17 cells (Fig. 4D and fig. S9E) and additionally through secretion of various chemokines like CCL2, CCL3, and CCL20 target the respective chemokine receptors on T_RM_17 cells (fig. S9E).

Next, we aimed to gain additional insight into intracellular signaling induced by CD40L, LTA, and GM-SCF in proinflammatory and profibrotic macrophages. For this, we used Kyoto Encyclopedia of Genes and Genomes (KEGG) pathways and annotated the DEG to the respective pathway by color-coding genes up-regulated in macrophages from patients with COVID-19 versus bacterial pneumonia. In proinflammatory macrophages, IL-1β, CXCL1, and CXCL8 might be produced because of signals transmitted by CD40, LTBR, and NFKB1. A similar signaling cascade could be induced in profibrotic macrophages. Furthermore, GM-CSF was associated with a pathway capable of triggering survival and proliferation signals in profibrotic macrophages (Fig. 4E).

Last, because it is known that CD4^+^ T cells are necessary for regulating the magnitude and quality of the cytotoxic CD8^+^ T cell response, we investigated the molecular mechanisms by which T_RM_17 might regulate the CD8^+^ T cell cytotoxic response in patients with COVID-19. The cytotoxic CD8^+^ T cell response has been proposed to mediate lung tissue damage in these patients (*12*, *13*). To identify highly cytotoxic CD8^+^ T cell clusters among the ones found (Fig. 2B), we created a cytotoxic scoring using proinflammatory genes that are expressed by cytotoxic T lymphocyte (CTL) in critical patients with COVID-19 as described by Chua *et al*. (*13*) and applied this dataset to the CD8^+^ clusters that we identified in our analysis. The highest cytotoxic signature was observed in CD8^+^ T_EM_ cluster 2 and CD8^+^ T_EM_ cluster 1 (Fig. 4F). We then calculated a second scoring to investigate whether CD8^+^ T cell clusters from the BALF of patients with COVID-19 might receive help from CD4^+^ T cells. To this end, we used the top 50 DEG identified by Ahrends *et al*. (*31*) as characteristic of T_EM_ cells receiving help from CD4^+^ T cells or not and applied this information to the different CD8^+^ T cell clusters from our dataset. We identified CD8^+^ T_EM_ cluster 2 to display the highest help module score (Fig. 4G). Next, to gain insight on how T_RM_17 cells and CD8^+^ T_EM_ affect each other, we determined the most specific and strongest interactions according to rank and connection strength. We identified CD70-CD27 and CCL5-CCR4 as the pathways highly engaged in this cell-to-cell interaction (Fig. 4H). Because CD4^+^-CD8^+^ T cell interaction occurs in a spatiotemporally organized interaction with DCs (*32*), we further dissected ligand-receptors interaction between T_RM_17 cells with DCs and CTL CD8^+^ T cells with DCs (Fig. 4I and fig. S9F). T_RM_17 cells had the potential to affect DCs via CD40L, FTL3LG, and GM-CSF (Fig. 4l). On the basis of all these data, we explored how the three cell populations could be connected and observed potential connections among T_RM_17, CD8^+^ CTL, and DCs (Fig. 4J).

In short, these data show the potential interaction of T_RM_17 cells and other tissue-specific immune cells, namely, macrophages and CTL CD8^+^, which have been associated with disease severity of COVID-19.

### The cellular map of GM-CSF–expressing cells

**Fig. 5:**
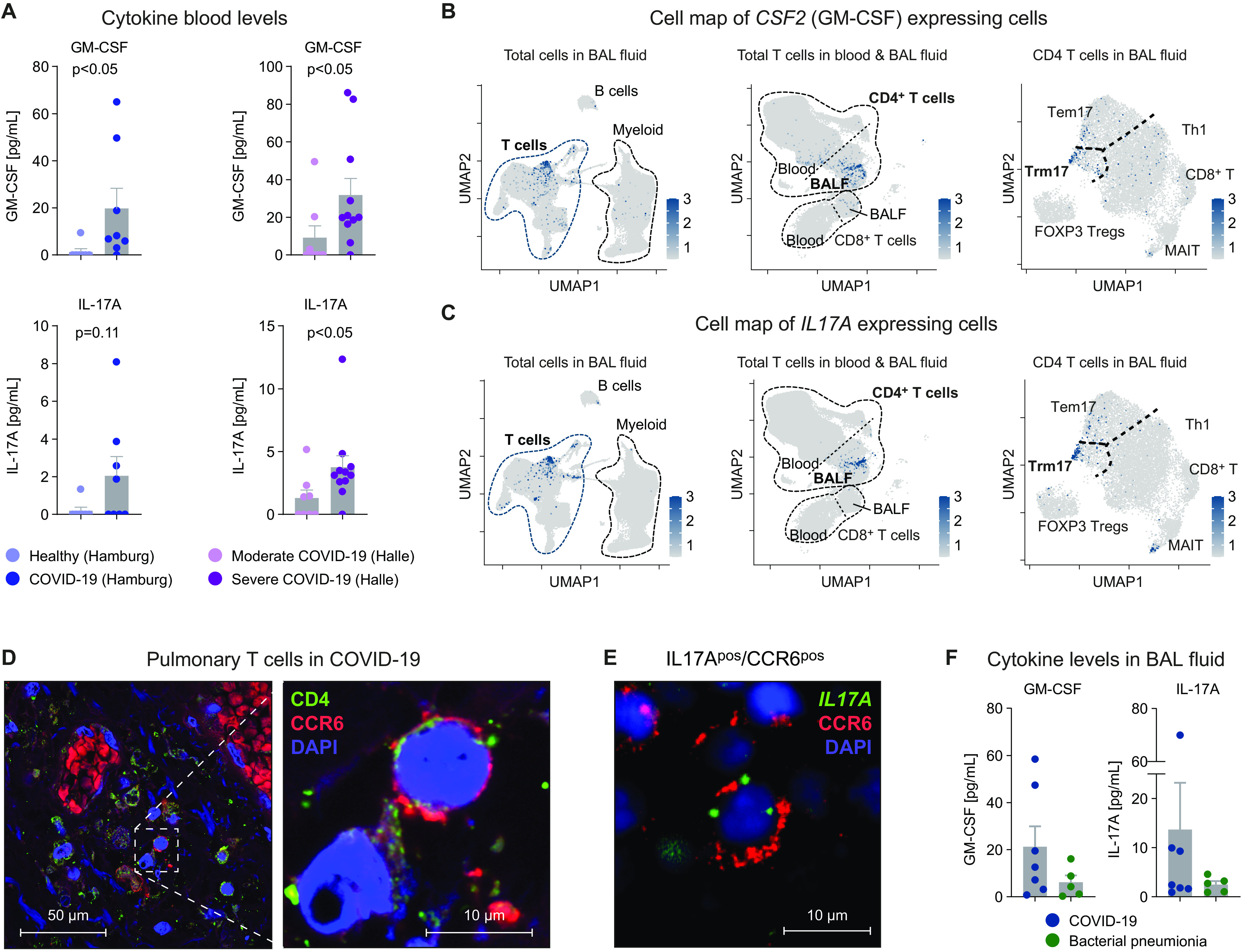
Cytokine secretion profile and cellular source of GM-CSF. (**A**) GM-CSF and IL-17A protein in serum of patients with COVD-19 (*n* = 8) and healthy controls (*n* = 7) from Hamburg and of patients with moderate (*n* = 8) or severe COVID-19 (*n* = 11) from Halle as indicated. Cell map of (**B**) *CSF2* (GM-CSF) expressing and (**C**) *IL17A* expressing cells (scale bars indicate normalized expression). Three different UMAPs with different cellular granularity showing the respective gene expression of in total cells of the BALF (left), total T cells of blood and BALF (middle), and total T cells in BALF (right) from all patients. (**D**) Immunofluorescence of CD4^+^ (green) CCR6^+^ (red) T_RM_17 cells in the lungs of a deceased patient with COVID-19 infection [nuclear staining 4′,6-diamidino-2-phenylindol (DAPI), blue] (two additional samples are presented in fig. S10B). (**E**) Combined immunofluorescence (CCR6) and FISH (*IL17A*) of lung samples from one patient with COVID-19. (**F**) Concentrations of the indicated cytokines in the BALF of patients with COVID-19 and bacterial pneumonia.

To test whether GM-CSF and IL-17A correlate with the severity of COVID-19, we first measured these two cytokines in the serum of patients with COVID-19 and of healthy blood donors and observed increased GM-CSF concentration in the patients (Fig. 5A). We excluded patient S9 from the Hamburg cohort because this patient received intravenous cytokine treatment. Second, we analyzed a different cohort, obtained from the University of Halle, which included patients with moderate and severe COVID-19 (*33*). We observed that GM-CSF and IL-17A appear to differentiate moderate versus severe disease (Fig. 5A).

To examine potential cellular sources of GM-CSF and IL-17A, we mapped *CSF2* (GM-CSF)– and *IL17A*-expressing cells on three UMAPs with different granularity and tissues (Fig. 5, B and C): all cells in the BALF (left), all T cells in BALF and peripheral blood (center), and CD4^+^ T cells in the BALF (right) and displayed gene expression in a violin/dot plot (overview, lung and blood; fig. S10A). These data show that gene expression of both cytokines is mainly restricted to the T_RM_17 cell subset from BALF.

Last, to further support the presence of T_RM_17 cells in the lungs of patients with COVID-19, we first performed immunofluorescence staining showing the presence of CD4^+^ cells expressing CCR6 in the perivascular infiltrate of the lungs (Fig. 5D and fig. S10B). Second, in a proof-of-principle analysis, the combination of immunofluorescence and fluorescence in situ hybridization (FISH) of CCR6 and *IL17A*, respectively, shows the presence of CCR6 and *IL17A* coexpressing cells in COVID-19 (Fig. 5E and fig. S10C). Third, we found high concentrations of GM-CSF and IL-17A as well as IFN-γ and IL-6 in the BALF of patients with COVID-19 (Fig. 5F and fig. S10D). These findings show that T_RM_17 cells are one potential source of the cytokines GM-CSF and IL-17A, which are prototypical of the hyperinflammation and are present locally within the lungs and in circulation in severe COVID-19.

## DISCUSSION

Here, we report a comprehensive single-cell transcriptional and TCR landscape of CD4^+^ T cells collected from the BALF and the peripheral blood of patients with severe COVID-19. We observed clonal expansion and characterized the activation profile of tissue-resident memory-like CD4^+^ T cells in the lungs of patients with COVID-19 that persist even after clearance of the virus. These cells express high amounts of the genes encoding the proinflammatory cytokines IL-17A/F and GM-CSF. Cell-cell interactome analysis uncovered a pathogenic network in the lung, involving GM-CSF–expressing T_RM_17 cells, IL-1β–expressing macrophages with a proinflammatory phenotype, macrophages expressing the GM-CSF receptor and genes associated with fibrosis, and cytotoxic CD8^+^ T cells. The relevance of our findings is further supported by the fact that serum protein levels of GM-CSF and IL-17A were elevated in a cohort of patients with severe COVID-19.

It has been speculated that reduction of T cells observed in the peripheral blood of patients with COVID-19 might be due to the recruitment of T cells to inflamed tissues (*34*). In support of this hypothesis, our flow cytometry data revealed an increased frequency of T cells in the lungs of these patients compared with patients with bacterial pneumonia while confirming reduced peripheral lymphocyte numbers. Moreover, activation status and cytokine expression were higher in lung T cells compared with peripheral T cells. By analyzing TCR clonality, we found a robust expansion of CD8^+^ T cells in the lungs of patients with COVID-19, as demonstrated by previous studies (*12*). However, the comparison with patients with bacterial pneumonia showed that this clonal expansion of CD8^+^ T cell subsets was a general hallmark of mild to severe lung inflammation. In contrast to this, CD4^+^ T cells and, in particular, those displaying a T_H_17 polarization state mainly expanded in the BALF of patients with COVID-19. One of these T_H_17 clusters expressed high levels of cytokines that have previously been associated with pathogenic activation of the immune system, such as GM-CSF (*35*, *36*) and IL-17A (*37*), as well as other known markers of T_H_17 cell pathogenicity, such as the transcriptional factor RBPJ (*20*).

Moreover, using TCR clonality analysis across compartments, we showed that clonally expanded cells in this T_H_17 cell cluster were almost exclusively present in BALF samples, but not in the peripheral blood. These data were supported by an enrichment of genes usually expressed by resident T cells and together provided evidence that these cells represent tissue-resident lung T_H_17 cells that are probably responsive to SARS-CoV-2–related antigens. In a previous study, we identified and characterized T_RM_17 cells in the lungs of mice and showed that they play a critical role in protecting from experimental *Klebsiella pneumoniae* infection (*15*). More recently, we found a correlation of kidney T_RM_17 cells and severity of immune-mediated kidney disease. Then, using mouse models, we demonstrated that T_RM_17 cells persist in the tissue after bacterial infection and can rapidly respond to inflammatory stimuli, such as IL-1β by producing IL-17A, which ultimately aggravates immune-mediated tissue injury (*16*). These two studies suggested that T_RM_17 cells can orchestrate a protective function against extracellular pathogens or fungi, but they can also participate in tissue damage if overactivated by inflammatory stimuli, such as IL-1β. In particular, because the T_H_17/IL-17 axis has not been linked to protective antiviral immunity, we propose that overproduction of IL-17A and GM-CSF by overactivated T_RM_17 cells is a feature of severe COVID-19 that might be involved in the immunopathology. However, our data do not rule out the possibility that T_RM_17 cells could provide a certain degree of protection at an early phase of the infection or in asymptomatic patients. Preclinical animal models of SARS-CoV-2 infection allowing the activity of T_RM_17-derived cytokines to be blocked are needed to fully address their role at different time points after the infection.

To provide a detailed view of how the different immune cell populations interact in COVID-19, we performed unbiased interactome analysis. Here, T_RM_17 cells were among the T cell subsets showing the strongest interaction with different myeloid cell subsets and CD8^+^ T cells. By ranking the interactions of T_RM_17 cells with myeloid cell subsets according to specificity and interaction strength, we identified GM-CSF, CD40L, and lymphotoxin-α as the most important effector pathways used by these cells to induce proinflammatory cytokine and chemokine production such as IL-1β, CXCL1, and CXCL8 in macrophages. Release of IL-1β by proinflammatory macrophages, in turn, could signal back to T_RM_17 cells to increase their pathogenicity (*16*, *38*). One of the most evident features of T_RM_17 cells was the expression of GM-CSF (see Figs. 2F and 5B), and the interaction with its receptor was among the top hits in the unsupervised interactome analysis of T_RM_17 cells with myeloid cell subsets. T cell–derived GM-CSF can result in activation and differentiation of myeloid cells (*39*). GM-CSF has further been shown to promote inflammatory tissue damage in a mouse model of Kawasaki disease, which is characterized by hyperinflammation that may share some features with severe COVID-19 (*40*). Enhanced frequencies of GM-CSF/IFN-γ coproducing T cells have been found in the blood of patients with COVID-19 and seemed to correlate with disease activity (*41*). Our data indicate that *CSF2*/GM-CSF–expressing cells are found in the lungs and coexpress *IL17A.* These data, in addition to the presence of CD4^+^CCR6^+^ T cells in the lung tissue as well as GM-CSF and IL-17A in the BALF, provide clinical evidence that T_RM_17 cell–associated cytokines are present in patients with severe COVID-19.

The major conclusions of this study derive from the comparison between cells taken from the blood and BALF of the patients with COVID-19. Nevertheless, in comparing patients with COVID-19 with patients with bacterial pneumonia, we observed that CD4^+^ T cells and, in particular, T_RM_17 cells were more clonally expanded in the BALF of the virally infected group. This comparison, however, also poses a key limitation of our study because we were unable to conclude whether the clonal expansion of T_RM_17 cells is specific to patients with COVID-19 or a common feature of severe viral infection. To address this point, we would have benefitted from having the BALFs from other viral infections such as influenza, in which the type of immune cells engaged is overall similar to a SARS-CoV-2 infection. A secondary limitation is our use of the term T_RM_17 cell, which was used on the basis of the expression profile and the reduced shared clonality between lung and blood of this population. However, the conclusion that these cells reside in the lungs is not definitive, as determination of tissue residency in human tissues remains challenging. Another limitation is the limited sample size of our study, and therefore, the results of this study need to be further validated in a larger cohort of patients, in which T_RM_17 cell associations with diseases severity are also examined. Almost all patients had severe COVID-19 according to the WHO classification in our study.

Last, on the basis of our data, we propose a model in which T_RM_17 cells are activated or reactivated as part of an ongoing cytokine storm, during which they can start producing proinflammatory cytokines such as GM-CSF. This could lead to further activation of macrophages and CD8^+^ T cells, which others have linked to the severity of the disease (*12*), and lastly mediate lethal lung damage (*36*, *39*). Two small pilot studies have indicated that targeting GM-CSF in patients with severe COVID-19 lung diseases using anti–GM-CSF receptor monoclonal antibodies mavrilimumab or lenzilumab, respectively, may be a strategy for improving clinical outcomes (*3*, *4*), although larger controlled clinical trials would be needed to determine efficacy and biological impact of such approaches. This network of tissue-resident cells may persist in the lungs even after the initiating event, e.g., viral infection, has been cleared, contributing to chronic lung pathology. In conclusion, our study provides a snapshot analysis of CD4^+^ T cells in the lungs of patients with severe COVID-19 and identifies T_RM_17 cells as one of the components of the lung-specific immune response. In addition, our data provide a rationale for investigating therapeutic approaches targeting T_RM_17 cells and the GM-CSF network in the search for urgently needed therapies for treating COVID-19 pneumonia.

## MATERIALS AND METHODS

### Study design

Patients with SARS-CoV-2 infection can develop a severe COVID-19 course with pulmonary involvement and high mortality. Because the adaptive immune system may play a major role in COVID-19 pathogenesis, we sought to investigate the immune response in the lungs of these patients by scRNA-seq with a focus on T cells and their cytokines. To this end, we planned simultaneous gene expression, TCR repertoire sequencing, and cell surface protein analyses. Because this is only possible from live cells, we obtained BALF from the lungs of patients with COVID-19 and from patients with bacterial pneumonia, which served as a control. We included nine patients with COVID-19 and five patients with bacterial pneumonia in the comprehensive scRNA-seq analysis at the University Medical Center Hamburg (tables S1 and S2). To compare blood cytokine levels in patients with moderate and severe COVID-19, we analyzed patients from the University of Halle, Germany (table S3).

### Cell isolation

Human BALF and peripheral blood for flow cytometry and scRNA-seq were both obtained from patients undergoing BAL. The indication and performance of bronchoscopy were in accordance with the current guideline recommendations (*42*). These studies were approved by the Ethik-Kommission der Ärztekammer Hamburg, local ethics committee of the chamber of physicians in Hamburg, and were conducted in accordance with the ethical principles stated by the Declaration of Helsinki. Informed consent was obtained from all participating patients or legal representatives. Single-cell suspensions were obtained from BALF by washing with phosphate-buffered saline followed by filtering through 100-, 70-, 40- (Greiner Bio-One, Kremsmünster, Austria), and 30-μm cell strainers (Partec, Görlitz, Germany). Leukocytes from blood samples were separated from red blood cells using BD Vacutainer CPT tubes with an integrated FICOLL gradient (BD Biosciences, San Jose, CA, USA). Samples were filtered through a 30-μm filter (Partec, Görlitz, Germany) before antibody staining and flow cytometry.

To minimize unspecific antibody binding, cells were incubated with Human BD FC Block (BD Biosciences) for 10 min. Next, cells were surface stained with fluorochrome conjugated antibodies [CD45 (clone HI30), CD3 (OKT3), CD4 (RPA-T4), CD8 (RPA-T8), CD56 (MEM-188), γδ-TCR 8(B1), CD31 (WM59), CD326 (9C4), CD14 (HCD 14), CD7 (CD7-6B7), CD16 (PC3G8), CD19 (HIB19), and CD324 (DECM-1); BioLegend and BD Biosciences], barcode-labeled antibodies (BioLegend) for 15 min (see table S4 for a complete list of antibodies and barcodes). Subsequently, a fixable dead cell stain (LIVE/DEAD Fixable Near-IR Dead Cell Stain Kit; Life Technologies, Carlsbad, CA) to exclude dead cells from analysis was used according to the manufacturer’s instructions. Cells were analyzed and sorted on a BD Biosciences FACS AriaFusion.

### Histology

For immunohistochemistry, human paraffin-embedded lung sections (2 μm) from patients with SARS-CoV-2 infection were stained with an antibody directed against CD3 (polyclonal rabbit anti-human, ref. A0452, DAKO, Glostrup, Denmark). Immunofluorescence microscopy was performed in 1-μm paraffin-embedded sections, after 15-min antigen retrieval with pH 9 antigen retrieval solution (Agilent, Santa Clara, CA, USA) and incubation with polyclonal primary goat anti-CD4 antibody (R&D Systems, Minneapolis, MN, USA, AF-379) and rabbit anti-CCR6 antibody (Abcam, Cambridge, UK, ab140768). Images were captured using a laser confocal microscope (LSM800, Zeiss, Jena, Germany).

For combined detection of *IL17A* mRNA and CCR6 FISH was performed on formalin-fixed paraffin-embedded human lung samples using RNAscope Technology as previously described (*43*) in accordance with the directions from Advanced Cell Diagnostics. The RNAscope Hs-IL17A-C3 probe from Advanced Cell Diagnostics (Advanced Cell Diagnostics, 310931-C3) was used as the target probe to detect IL-17a mRNA. Fluorescent labeling of the target probe was performed using OPAL 690 dye (dilution, 1:1000; Akoya Biosciences, FP1497001KT). Subsequent immunofluorescence labeling was performed with an antibody against CCR6 (dilution: 1:200; OriGene Technologies, TA316610) in the same sections after completing the FISH protocol. Epifluorescence imaging was performed using the THUNDER Imager 3D Live Cell and 3D Cell Culture (Leica Microsystems).

### Multiplex

We used a bead-based immunoassay technology (LEGENDplex, BioLegend) to quantify the concentration of cytokines in the serum and BALF for each sample. The premixed Human Anti-Virus Response Panel (catalog no. 740349) and the Human Essential Immune Response Panel (catalog no. 740929) were applied to analyze the relevant cytokines following the manufacturer’s protocol. Values below the limit of detection were considered zero. Collection of the Halle cohort was performed under institutional review board approval numbers 2020-039 and 11/17. This cohort is partially published (*33*).

### Cell sorting, library preparation, and next-generation sequencing

To enrich for T cells from the BALF, we FACS-sorted T cells, alveolar macrophages, monocytes, CD45^high^CD3^neg^ cells (including innate lymphoid cells), and CD45^neg^ cells (lung cells) according to the gating strategy presented in fig. S1A. From peripheral blood, we FACS-sorted CD3^pos^ T cells. Subsequent scRNA-seq using the 10X Chromium Controller (10X Genomics, Pleasanton, CA, USA) was loaded with the following proportions: first lane: 100% BALF T cells; second lane: BALF 17% alveolar macrophages, 17% monocytes, 33% CD45^neg^ cells, and 33% CD45^high^CD3^neg^ cells; third lane: 100% blood T cells (cell numbers were based the FACS information).

Single-cell libraries were generated with the 10X Genomics Chromium Single Cell 5′v1.1 reagents kit according to the manufacturer’s instructions. Fifty-nanometer cDNA was used for gene expression library construction. Quality control (QC) was performed with hsDNA Qubit (Thermo Fisher Scientific, Waltham, MA, USA) and BioAnalyzer (Agilent). The libraries were sequenced on an Illumina NovaSeq 6000 system (S4 flow cell) with 150 base pairs and paired-end configurations.

### Preprocessing of single-cell RNA-seq and CITE-seq data

The Cell Ranger software pipeline (v3.1.0, 10X Genomics) was used to demultiplex cellular barcodes and map reads to the human reference genome (refdata-cellranger-GRCh38-3.0.0) (command cellranger count). The CITE-seq antibody and barcode information was included in a feature reference csv file and passed to the cellranger count command. As the output, we obtained the feature-barcode matrix that contains gene expression counts alongside CITE-seq counts for each cell barcode. The feature-barcode matrices for all the sample were further processed by the R package Seurat (v3.1.4) (*44*). As a QC step, we first filtered out the cells in which less than 200 genes were detected in the BALF samples and less than 500 genes were detected in the blood samples. To remove potential doublets, we excluded cells with total number of detected genes more than 5000. After visual inspection of the distribution of cells by the percentage of mitochondrial genes expressed, we further removed low-quality cells with more than 5% mitochondrial genes of all detected genes. We used LogNormalize method in Seurat to normalize the scRNA-seq and CITE-seq counts for the cells passed the QC.

### Sample aggregation and integration

For the BALF cell analysis, we first aggregated the BALF CD3^+^ sample and CD3^−^ sample for each patient using the function merge in Seurat. (We excluded the CD3^−^ samples of patients S2 and B3 and the CD45^−^ sample of patient S9 due to low sequence quality. For patient S9, we merged the CD45^+^ sample and EpCAM^+^ sample). After we obtained the merged BALF Seurat object for each patient, to remove the batch effects across different patients, we applied the integration method implemented in Seurat (function FindIntegrationAnchors and IntegrateData, dims = 1:30). For the blood CD3^+^ cell analysis, we directly applied the integration to the samples of all patients. For the combined analysis of BALF and blood T cells, we selected T cell clusters identified in the BALF samples (as described below) and aggregated with corresponding blood CD3^+^ samples for each patient using the merge function. Integration was then applied to the merged objects for all the patients.

### Dimensionality reduction and clustering

For each integrated object, the integrated matrix was scaled by ScaleData function (default parameters) and highly variable genes were detected (function FindVariableFeatures, selection.method = “vst”, nfeatures = 2000). Principal components analysis was performed on the scaled data (function RunPCA, npcs = 30) to reduce dimensionality. Thirty principal components were used to compute the *k*-nearest neighbor graph on the basis of the Euclidean distance (function FindNeighbors), which then generated cell clusters using function FindClusters. The resolution parameter of the FindClusters function for each dataset was also determined by exploration of top marker genes of each clusters. UMAP was used to visualize clustering results. The top DEG in each cluster was found using the FindAllMarkers function (min.pct = 0.25 and logfc.threshold = 0.25) that ran Wilcoxon rank sum tests. Seurat functions AverageExpression and DoHeatmap were used to visualize the expression of the top marker genes or CITE-seq protein expression in each cell cluster. The top marker genes as well as the CITE-seq expression patterns were then used to determine the cell type of each cluster. The differential expression between selected clusters were calculated by the FindMarkers function (min.pct = 0.1), which also ran Wilcoxon rank sum tests.

### BALF T cells, myeloid cells reintegration, and subclustering

For the separate analysis of BALF T cells and BALF myeloid cells, we selected the clusters identified in the total BALF samples and reintegrated them by patients. Reclustering was performed after integration as described above and a detailed cell-type annotation was obtained after exploring the top marker genes and the CITE-seq expression profiles of clusters. For the subclustering analysis of CD4^+^ cells, reintegration by patients and reclustering were also performed respectively before the identification of the cell subtypes.

### Processing of TCR-seq data and integration

TCR-seq data for each sample were assembled by the Cell Ranger software (v3.1.0, 10X Genomics) with the command cellranger vdj using the reference genome (refdata-cellranger-vdj-GRCh38-alts-ensembl-3.1.0). For each sample, Cell Ranger generated an output file, filtered_contig_annotations.csv, containing TCR-α chain and TCR-β chain CDR3 nucleotide sequences for single cells that were identified by barcodes. The R package scRepertoire (v1.2.1) (*45*) was used to further combine the contig_annotation data of different samples to a single list object (function combineTCR). The combined TCR contig list file was then integrated with the corresponding Seurat object of the scRNA-seq data using the function combineExpression (cloneCall = “gene+nt”). Only the cells with both TCR and scRNA-seq data were kept for downstream clonotype analysis. The clonotype was defined according to the genes comprising the TCR and the nucleotide sequence of the CDR3 region. The frequency of the each clonotype in each patient was then calculated as clone count. To get a normalized clone count size for each clonotype, we also calculated the clone size proportion (clone count divided by number of cells per patient). The clone count and clone size proportion were added to metadata of the single-cell matrices.

### Calculation of gene signature scores

Signature scores of gene sets were calculated by Seurat function AddModuleScore (nbin = 24 and ctrl = 100). The cytokine secretion gene set includes major proinflammatory cytokines produced by T cells. The residency and migration gene sets were obtained from a core list of up-regulated and down-regulated genes by CD4 tissue-resident T cells (table S5) (*16*, *46*).

### Cell-cell interaction analysis

We applied CellphoneDB’s statistical analysis method (2.1.2) and receptor-ligand database (2.0.0) to calculate statistically enriched cell-cell interactions (https://github.com/Teichlab/cellphonedb). We used the log-normalized RNA assay of our BALF dataset containing all samples and selected cells either from patients with COVID-19 or patients with bacterial pneumonia to gain the count matrix. Because we have different levels of subclustering, we annotated each cell according to its cluster in its deepest level and used it as metadata input (clustering level: all BALF samples > T cells > CD4+ T cells; all BALF samples > myeloid cells). We ran CellphoneDB with the default parameters. In total, CellphoneDB returned 13,034 significant (*P* < 0.05) interactions. The rank of a ligand-receptor pair was calculated by CellphoneDB dividing its total number of significant *P* values by the number of cluster-cluster comparisons. For downstream analysis, we excluded integrin ligand-receptor pairs and interactions being annotated as not secreted. Moreover, we excluded CCL20-CXCR3 interactions (Id_cp_interaction “CPI-SS0F8C664D9”) because of a lack of evidence in the literature.

### Connection strength

The connection strength of a specific interaction between two clusters was calculated by multiplying the mean expression of the ligand in the ligand cluster by the proportion of cells expressing the receptor in the receptor cluster. The receptor was expressed if the log-normalized expression value was greater than 0. In case of a receptor complex, the receptor component expressed in the least cells was used.

### Pathway analysis

The differential expression of SARS-CoV-2–infected patients was calculated using patients with bacterial pneumonia as control group. With the differential expression data, enriched pathways were determined using Gene Ontology terms and KEGG pathways. This reveals commonly known pathways (e.g., Janus kinase–signal transducer and activator of transcription signaling and phosphatidylinositol 3-kinases pathway). From these results, relevant parts of the pathways were curated and combined to the final pathway, using the KEGG Markup Language schema (www.kegg.jp/kegg/xml/docs/). Coherent components of these enriched pathways were combined to a single representative pathway for each subset of macrophages. The log fold change of DEG was added as color code to the elements of the pathway.

### Network plots

Network plots were created using the R package igraph (1.2.5) (https://github.com/igraph). The layout of the network in Fig. 4A was calculated using the Fruchterman-Rheingold algorithm (function layout_with_fr, niter = 5000). The weight parameter was set to the number of interactions between two clusters. The clusters “CD8_Tcells,” “M1_HSP,” and “epithelial cells” were excluded. The vertex size was set by using graph strength, which sums up the edge weights for each vertex. The curated interaction map in Fig. 4D was made using the R package igraph (https://github.com/igraph) without applying the Fruchterman-Reingold algorithm. The thickness of the lines correlates with the respective connection strength ([average ligand expression] × [proportion of cells expressing the receptor]).

### Statistics

Statistical analysis was performed using GraphPad Prism (La Jolla, CA). The results are shown as single data points with the means ± SEM in a scatter dot plot. Differences between two individual groups were compared using a Mann-Whitney test. In the case of three or more groups, Wilcoxon test was used.

## SUPPLEMENTARY MATERIALS

immunology.sciencemag.org/cgi/content/full/6/56/eabf6692/DC1 Fig. S1. Sorting strategy and clustering information of BALF immune cells from all patients. Fig. S2. Clustering information of blood T cells from all patients. Fig. S3. Flow cytometry of cells from peripheral blood and BALF. Fig. S4. Location of T cells in the lungs of COVID-19. Fig. S5. Clustering information of BALF T cells from all patients. Fig. S6. Clonal expansion analysis of BALF T cells from all patients. Fig. S7. Subclustering of clonally expanded BALF CD4 T cells from all patients. Fig. S8. Clustering information of BALF myeloid cells from all patients. Fig. S9. Cell-cell interaction of T cells with myeloid cells and epithelial cells. Fig. S10. Cytokine levels in BALF. Table S1. Baseline characteristics and disease-related parameters of patients with COVID-19 and controls. Table S2. Relevant medication of patients with COVID-19 and controls. Table S3. Baseline characteristics of the Halle cohort. Table S4. CITE-seq antibodies and barcodes (Excel file). Table S5. Gene sets (Excel file). Table S6. Raw data file (Excel file).

## Appendix

View/request a protocol for this paper from *Bio-protocol*.
